# Supplementary material for: Efficient conversion of propane in a microchannel reactor at ambient conditions
Source: Nat Commun. 2024 Jan 29;15:884. doi: 10.1038/s41467-024-45179-1 (PMC10825187; doi:10.1038/s41467-024-45179-1)
Supplement: Supplementary file 1 — Supplementary Information [file 41467_2024_45179_MOESM1_ESM.pdf]

Supplementary Information

Efficient conversion of propane in a microchannel reactor at ambient conditions

Chunsong Li[1,4], Haochen Zhang[1,4], Wenxuan Liu[1], Lin Sheng[1], Mu-Jeng Cheng[2], Bingjun Xu[3], Guangsheng Luo[1], Qi Lu[1]

[1]State Key Laboratory of Chemical Engineering, Department of Chemical Engineering, Tsinghua University, Beijing 100084, China.

[2]Department of Chemistry, National Cheng Kung University, Tainan 701, Taiwan.

[3]College of Chemistry and Molecular Engineering, Peking University, Beijing 100871, China.

[4]These authors contributed equally: Chunsong Li and Haochen Zhang.

Email: gsluo@tsinghua.edu.cn (Guangsheng Luo); luqicheme@mail.tsinghua.edu.cn (Qi Lu)

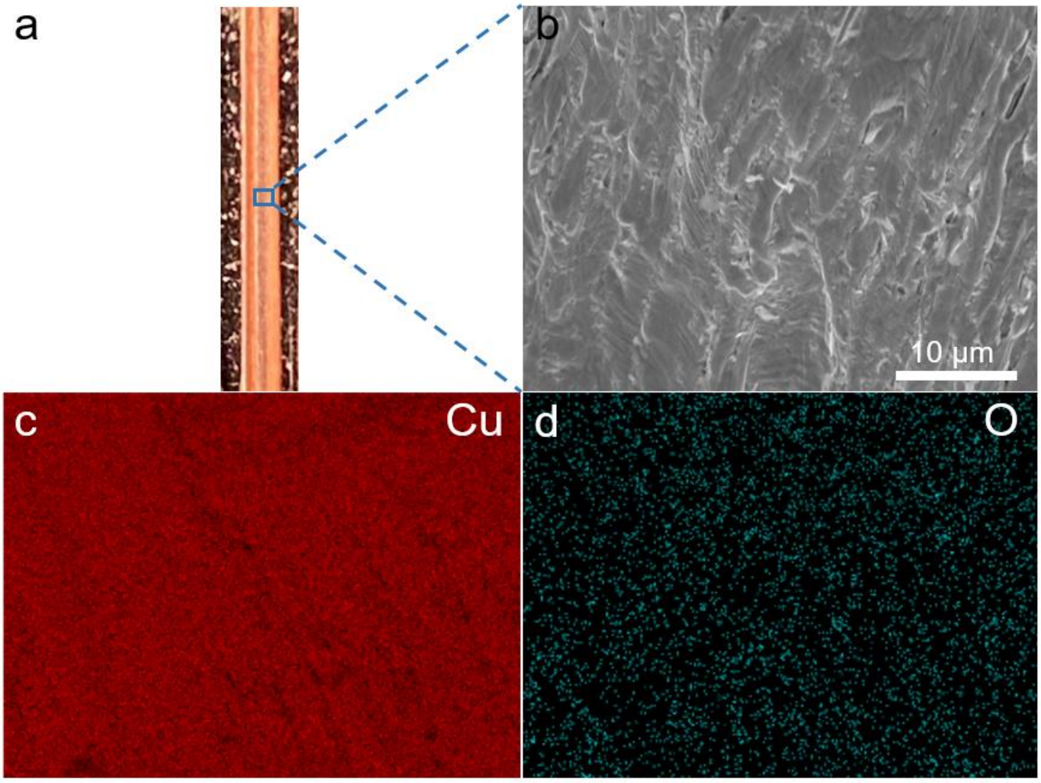

Supplementary Figure 1. (a) A photo of cross section of the Cu microtube, (b-d) SEM image (b) with the elemental distribution by EDX for Cu (red) (c) and O (cyan) (d).

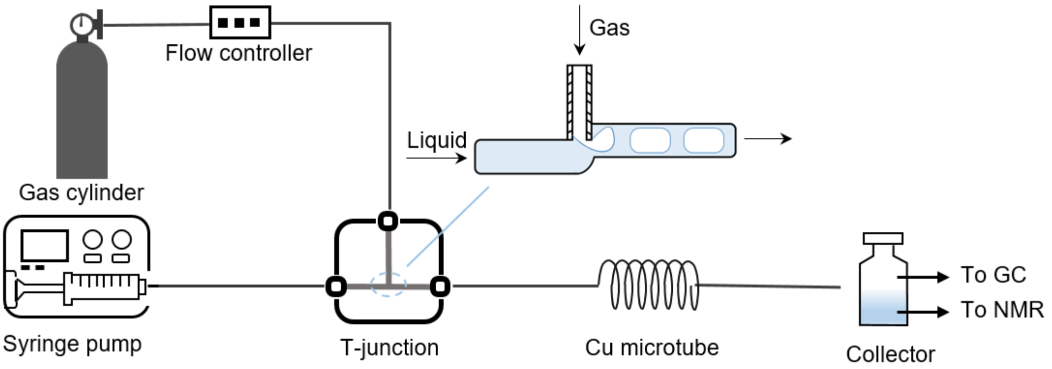

Supplementary Figure 2. Scheme of the experimental setup for propane activation in Cu microchannel reactor.

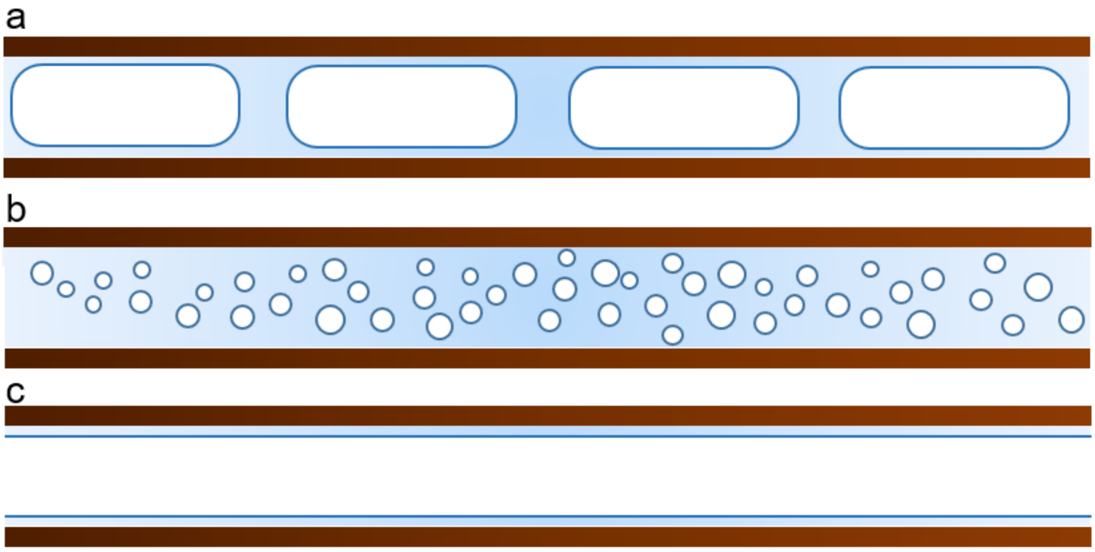

Supplementary Figure 3. Comparison of flow patterns of (a) Taylor flow, (b) bubbly flow and (c) annular flow.

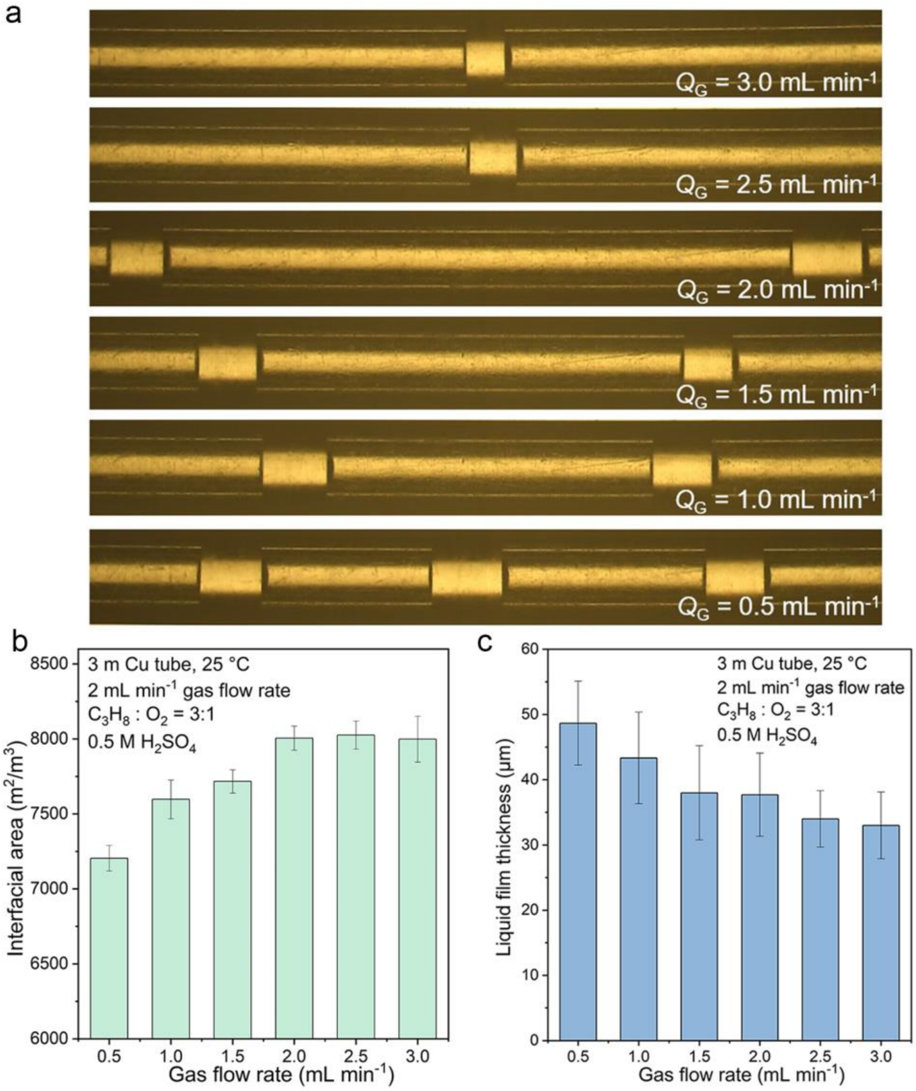

Supplementary Figure 4. (a) High-speed camera photographs of the gas-liquid Taylor flow at various gas flow rates (Q_G) in a transparent polypropylene tube with identical diameter as Cu tube. The total liquid flow rate (Q_L) was fixed at 0.2 mL min^{-1}. (b) Gas-interfacial area calculated from gas-liquid flow pattern at various gas flow rates with a fixed liquid flow rate of 2 mL min^{-1}. (c) Thickness of the liquid film calculated from gas-liquid flow pattern at various gas flow rates with a fixed liquid flow rate of 0.2 mL min^{-1}.

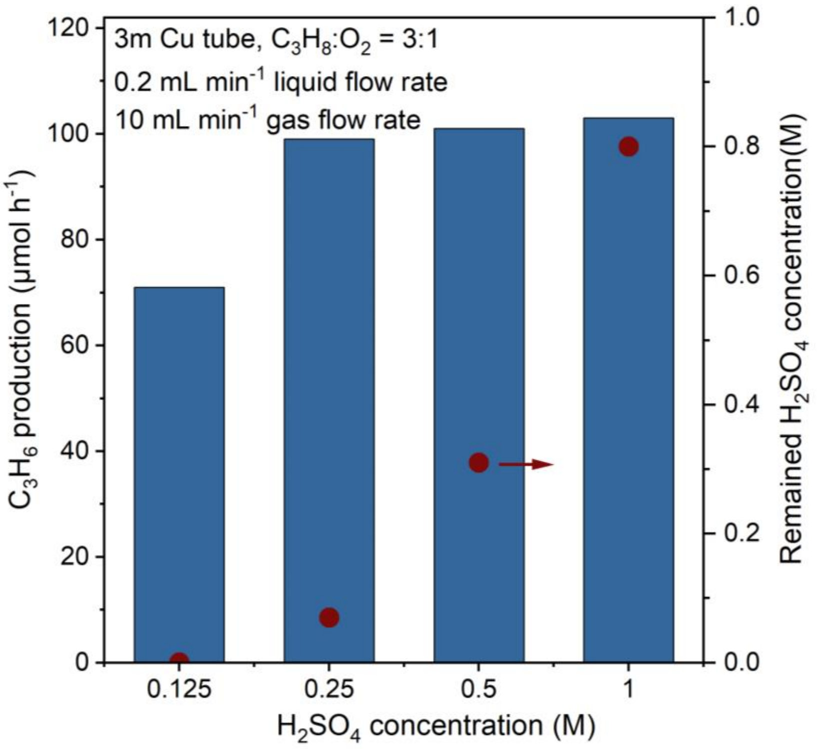

Supplementary Figure 5. Dependence of propane oxidation rate on sulfuric acid concentration.

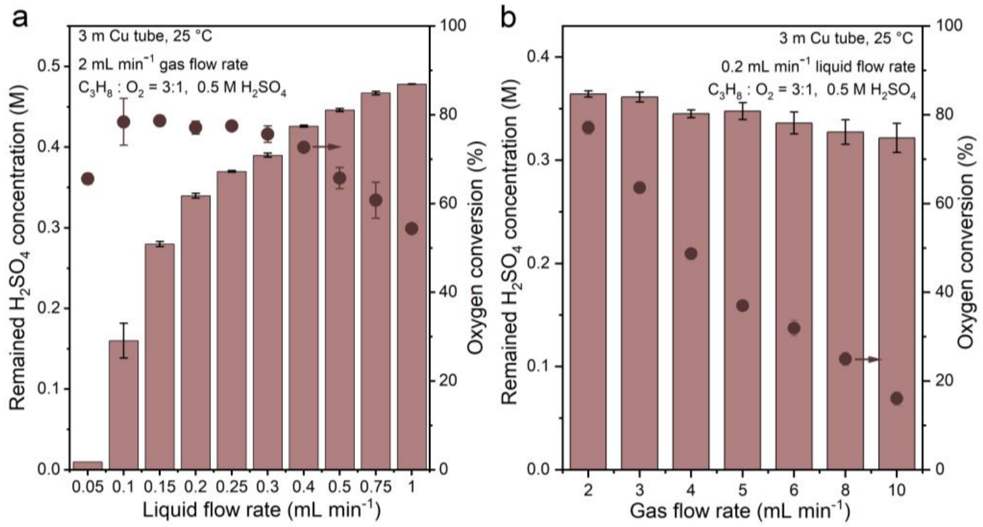

Supplementary Figure 6. The remaining sulfuric acid concentration and oxygen conversion rate after reaction at (a) various liquid flow rates with a fixed total gas flow rate of 2 mL min^{-1} and (b) various gas flow rates with a fixed liquid flow rate of 0.2 mL min^{-1}. The initial concentration of sulfuric acid is 0.5 M.

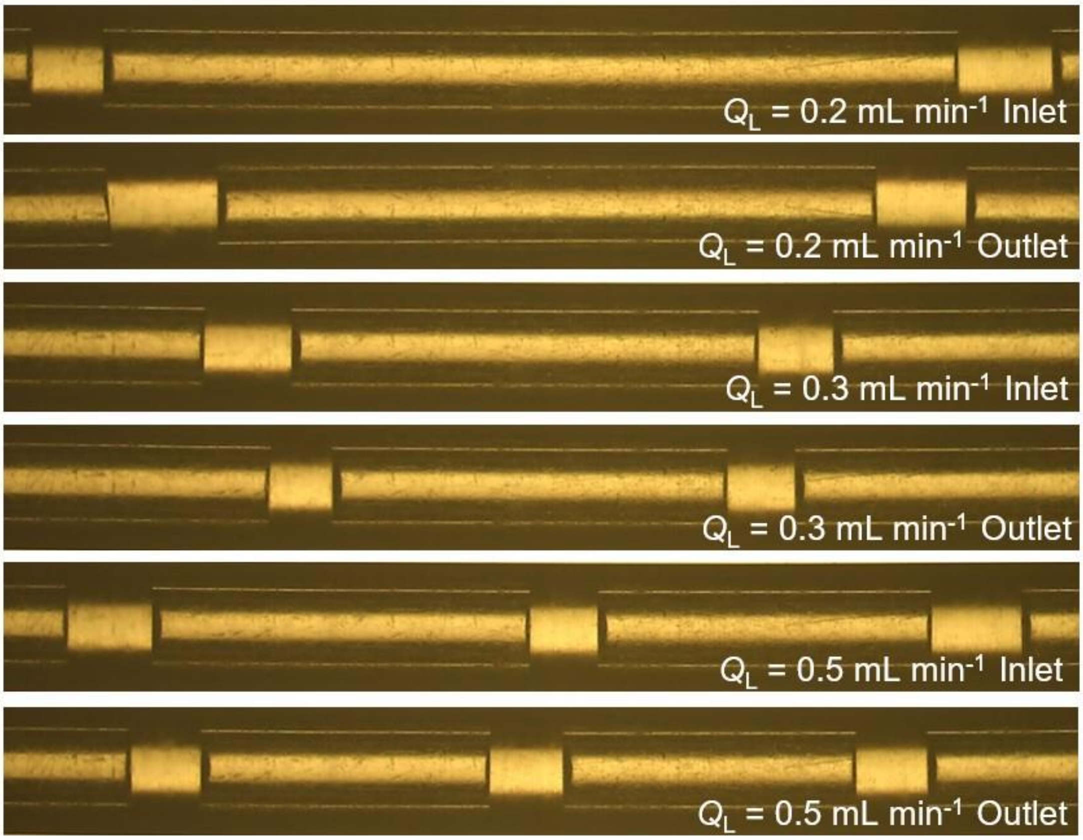

Supplementary Figure 7. Comparison of the high-speed camera photographs of the Taylor flow between inlet and outlet at various liquid flow rates (Q_L). The gas flow rate (Q_G) was fixed at 2 mL min^{-1}.

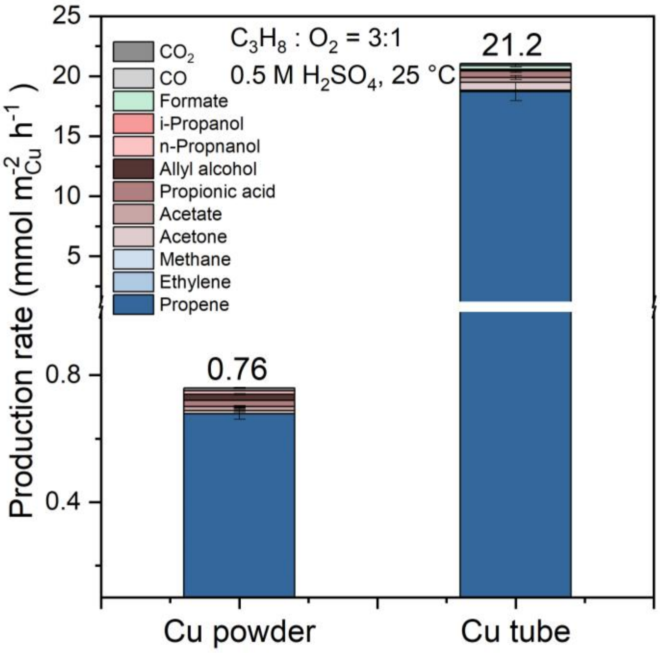

Supplementary Figure 8. Comparison of production rates of propane oxidation conducted on Cu powders and Cu tubes.

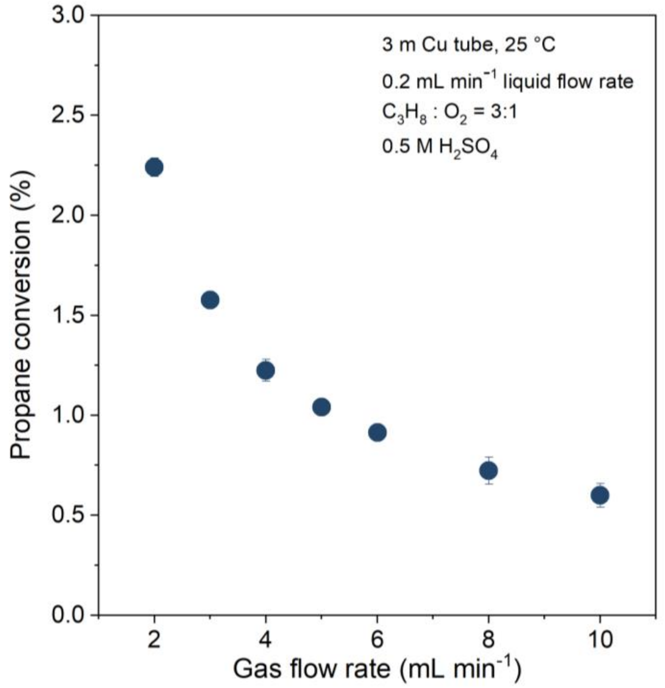

Supplementary Figure 9. Propane conversion rate at various gas flow rates with a fixed liquid flow rate of 0.2 mL min^{-1}.

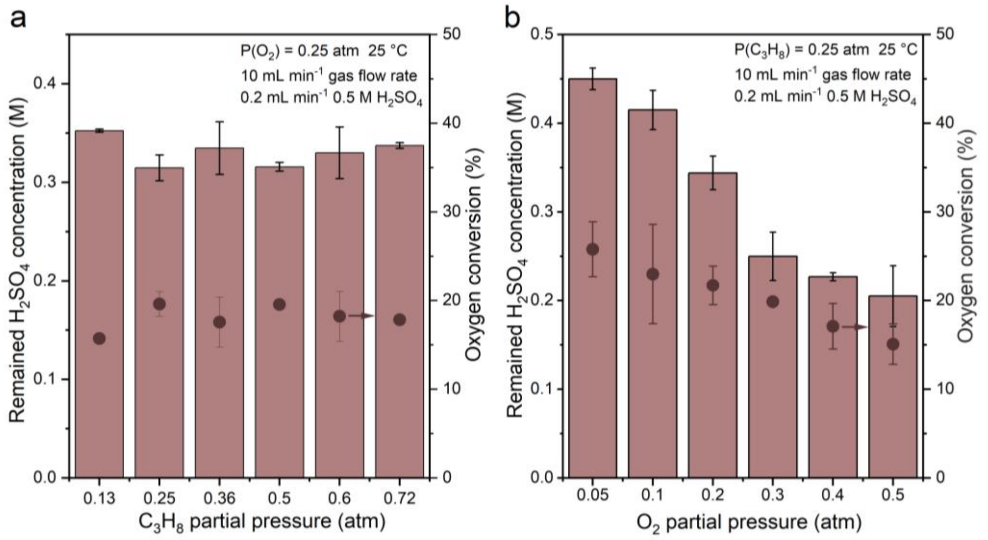

Supplementary Figure 10. The remaining sulfuric acid concentration and oxygen conversion rate after reaction at (a) various propane partial pressures and (b) various propane partial pressures. The initial concentration of sulfuric acid is 0.5 M.

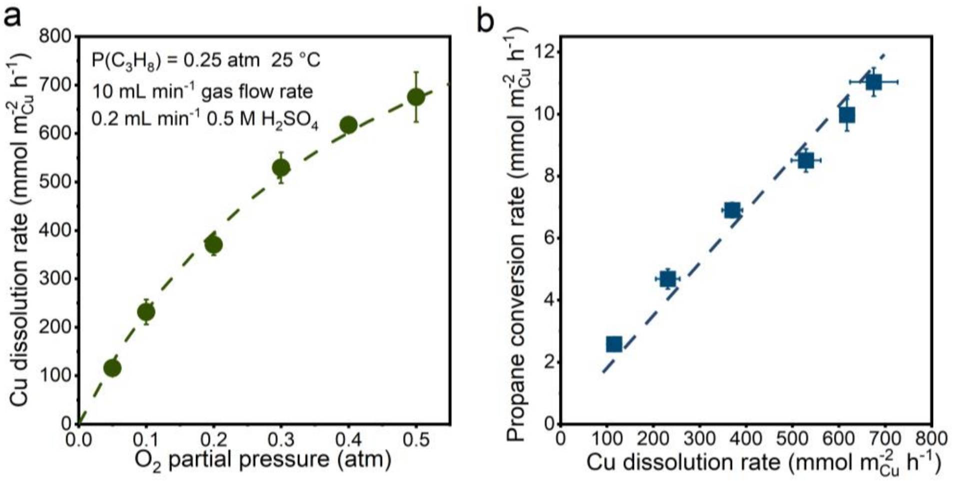

Supplementary Figure 11. The relationship between (a) Cu dissolution rate and O_2 partial pressure, (b) propane conversion rate and Cu dissolution rate.

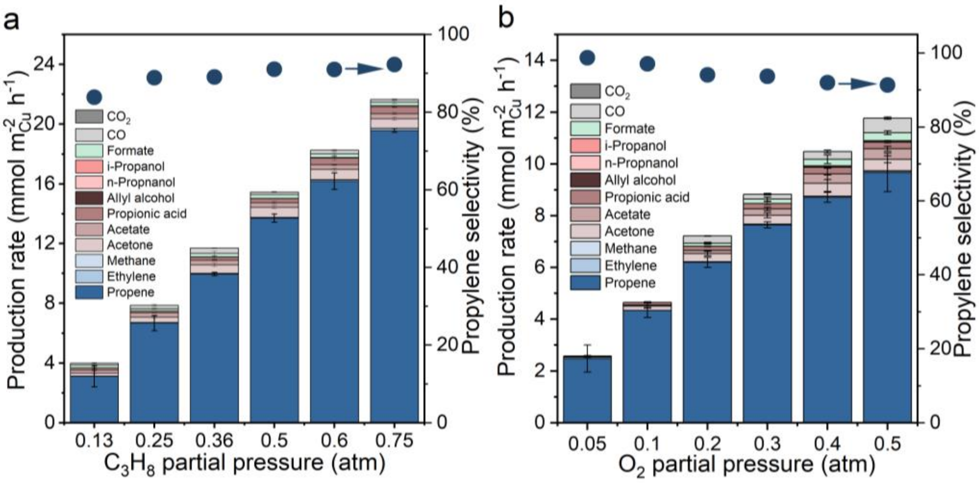

Supplementary Figure 12. Production rates of propane oxidation at (a) various propane partial pressures and (b) various O_2 partial pressures.

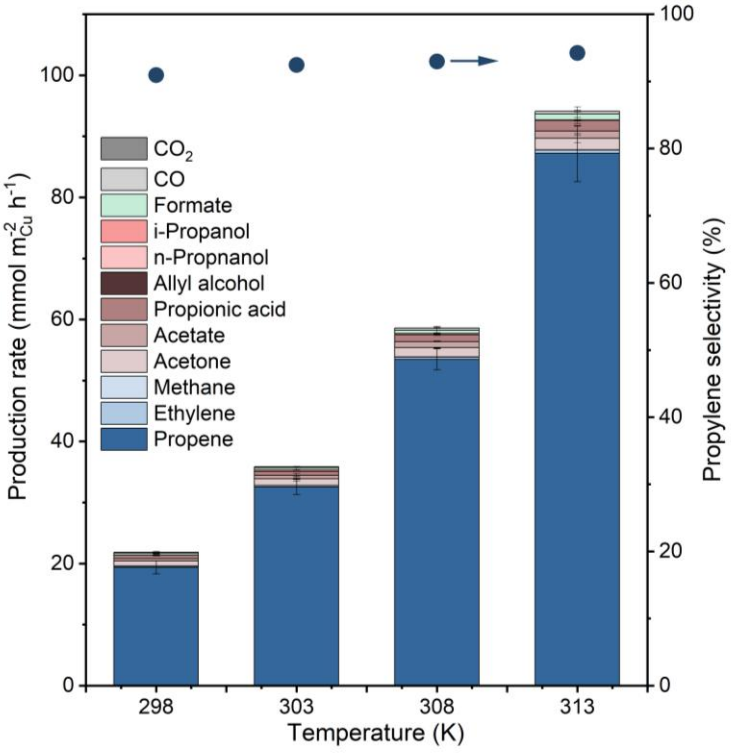

Supplementary Figure 13. Product distribution of propane conversion at elevated temperatures.

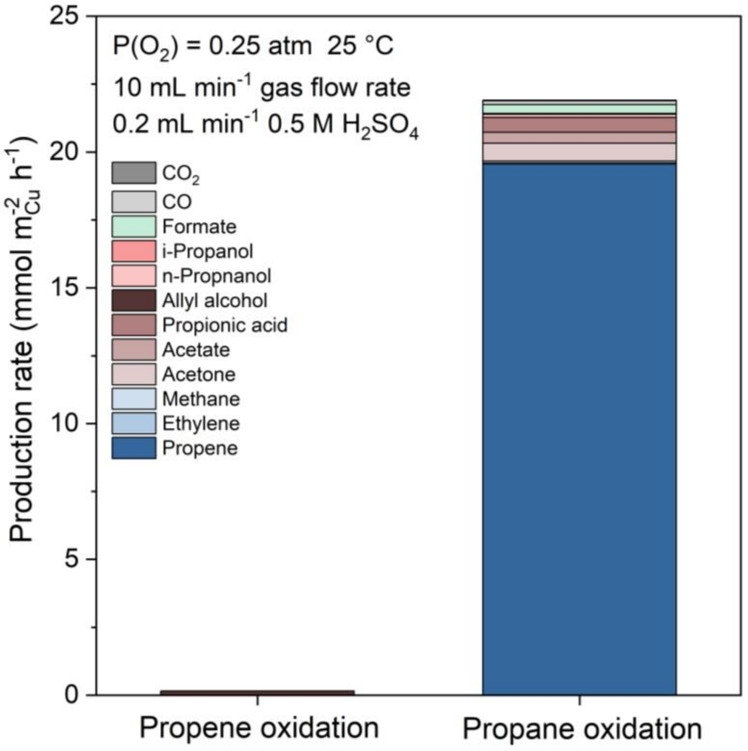

Supplementary Figure 14. Comparison of production rates for main products from propene oxidation and propane oxidation.

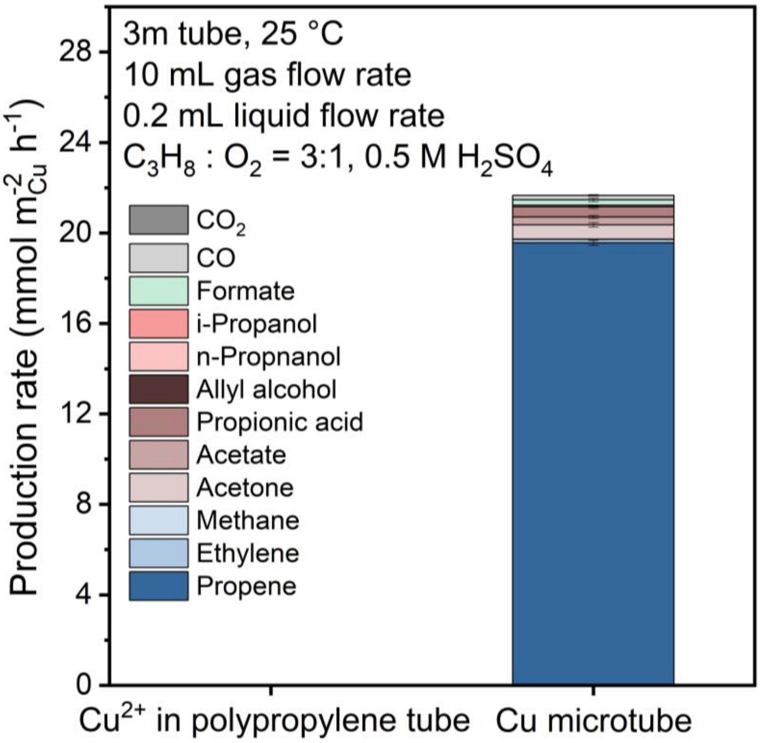

Supplementary Figure 15. Propane activation of using polypropylene tube instead Cu microtube containing 0.2 M $CuSO_4$ and 0.5 M H_2SO_4 in the liquid flow.

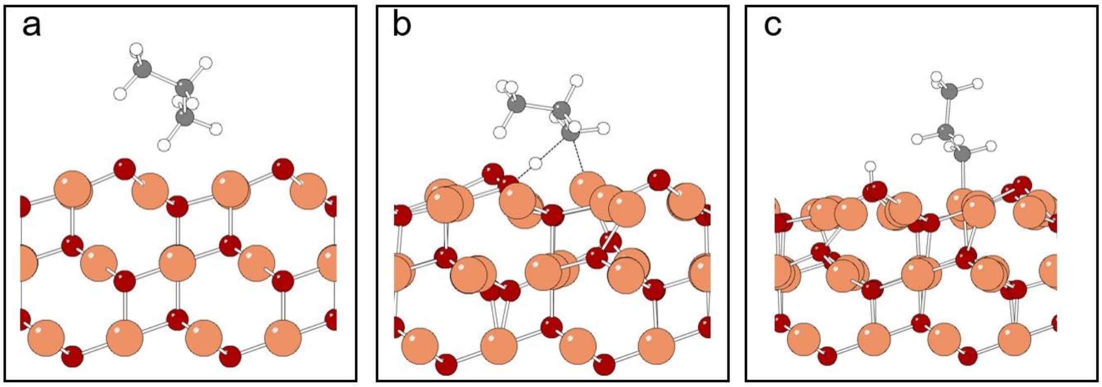

Supplementary Figure 16. Initial state (a), transition state (b), and final state (c) of initial primary C−H bond cleavage of propane on Cu oxide surface.

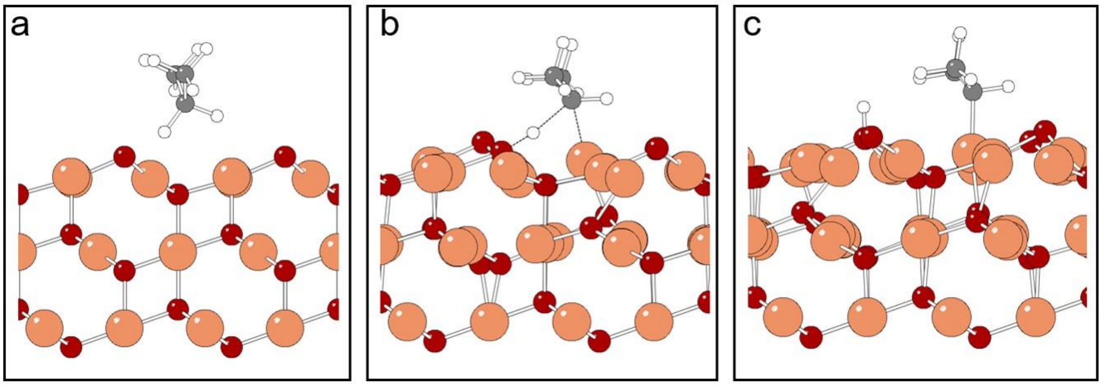

Supplementary Figure 17. Initial state (a), transition state (b), and final state (c) of initial secondary C−H bond cleavage of propane on Cu oxide surface.

Supplementary Table 1

Comparison of C_3H_6 production rates in propane activation

Catalyst	Temperature (°C)	Production rates of C_3H_6 (mmol $m^{-2}_{cat.}$ h^{-1})
This work	25	19.57
	30	32.6
	35	53.5
	40	87.2
Cu powder[1]	25	0.714
NiO[2]	500	1.893
BNNT[3]	490	1.296
CeO_2[4]	500	1.1
h-BN[3]	490	0.396
h-BN/In_2O_3-S[5]	520	0.635
VO_x/SiO_2[6]	525	0.196
Cr-TiO_2[7]	540	0.077
Mo/$SmVO_4$[8]	550	0.075
Mo_1V_6[9]	500	0.0648
SS-BNNSs[10]	510	0.055
VO_x/BN-T[11]	600	0.034
NbO_x/CeO_2[12]	400	0.02
CMK-3[13]	600	0.0078
BS-1[14]	480	0.0033

Supplementary Note 1

1. Thickness of liquid film and gas-liquid interfacial area calculation.

To calculate the liquid film thickness (δ) between the gas bubble and Cu surface, two parameters of the gas bubble length (L_b) and liquid slug length (L_s) are defined, as shown in Supplementary Figure 18, and they can be measured from the recorded video.

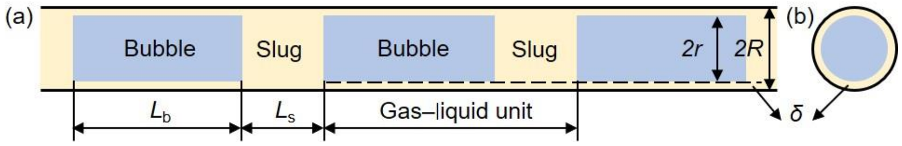

Supplementary Figure 18. Schematic diagram of the gas–liquid flow in microchannel

Regarding the radius of the cross-sectional area of the bubble as r; thus, the volume of the gas bubble (V_b), liquid slug (V_s), and liquid film (V_δ) in a single gas–liquid unit is calculated as Eqs. (1–3):

$$V_b = \pi r^2 L_b \tag{1}$$

$$V_s = \pi r^2 L_s \tag{2}$$

$$V_\delta = \pi\left(R^2 - r^2\right)\left(L_b + L_s\right) \tag{3}$$

Due to the leakage flow of the liquid phase in the gutter between the gas bubble and the channel wall, the gas–liquid flow state will deviate from the ideal plug flow. Ali et al. reported that the actual gas holdup (α) in the microchannel is proportional to the volumetric fraction of the gas phase (β) at the inlet of the microreactor, and the coefficient is 0.8[15]. The calculation methods of α and β are shown in Eq. (4). Q_G and Q_L are the gas flow rate and liquid flow rate, respectively. V_G is the gas volume in a single gas–liquid unit, V_L is the total liquid volume in a single gas–liquid unit and equals the sum of liquid slug volume and liquid film volume.

$$\alpha = \frac{V_G}{V_G + V_L} = 0.8\frac{Q_G}{Q_G + Q_L} = 0.8\beta \tag{4}$$

$$V_L = V_s + V_\delta = \pi\left(R^2 - r^2\right)L_b + \pi R^2 L_s \tag{5}$$

$$V_G = V_b = \pi r^2 L_b \tag{6}$$

Therefore, the parameter of r can be determined via Eqs. (1–6), and the liquid film thickness (δ) can be obtained via Eq. (7).

$$\delta + r = R \tag{7}$$

The gas bubble surface area (S) can be calculated via Eq. (8)

$$S = 2\pi r L_b + 2\pi r^2 \tag{8}$$

The gas-liquid interfacial area (a) can be determined by Eq. (9)

$$a = \frac{S}{V_b + V_s + V_\delta} \tag{9}$$

Supplementary References

1. Zhang, H. et al. Activation of light alkanes at room temperature and ambient pressure. *Nat. Catal.* **6**, 666-675 (2023).
2. Li, J.-H. et al. Mesoporous nickel oxides as effective catalysts for oxidative dehydrogenation of propane to propene. *Appl. Catal., A: Gen.* **382**, 99-105 (2010).
3. Grant, J. T. et al. Selective oxidative dehydrogenation of propane to propene using boron nitride catalysts. *Science* **354**, 1570-1573 (2016).
4. Xie, Q. et al. Oxidative Dehydrogenation of Propane to Propylene in the Presence of HCl Catalyzed by CeO_2 and NiO-Modified CeO_2 Nanocrystals. *ACS Catal.* **8**, 4902-4916 (2018).
5. Cao, L. et al. Antiexfoliating h-BN superset In_2O_3 Catalyst for Oxidative Dehydrogenation of Propane in a High-Temperature and Water-Rich Environment. *J. Am. Chem. Soc.* **145**, 6184-6193 (2023).
6. Barman, S. et al. Single-Site VO_x Moieties Generated on Silica by Surface Organometallic Chemistry: A Way To Enhance the Catalytic Activity in the Oxidative Dehydrogenation of Propane. *ACS Catal.* **6**, 5908-5921 (2016).
7. Ma, F., Chen, S., Wang, Y., Chen, F. & Lu, W. Characterization of redox and acid properties of mesoporous Cr–TiO_2 and its efficient performance for oxidative dehydrogenation of propane. *Appl. Catal., A: Gen.* **427-428**, 145-154 (2012).
8. Barbero, B. Molybdenum role: Mo-Sm-V-O catalytic system for propane oxidative dehydrogenation. *Appl. Catal., A: Gen.* **252**, 133-147 (2003).
9. Chen, S. et al. Modulating Lattice Oxygen in Dual-Functional Mo-V-O Mixed Oxides for Chemical Looping Oxidative Dehydrogenation. *J. Am. Chem. Soc.* **141**, 18653-18657 (2019).
10. Cao, L. et al. Spherical Superstructure of Boron Nitride Nanosheets Derived from Boron-Containing Metal-Organic Frameworks. *J. Am. Chem. Soc.* **142**, 8755-8762 (2020).
11. Jiang, X. et al. Multiple Promotional Effects of Vanadium Oxide on Boron Nitride for Oxidative Dehydrogenation of Propane. *JACS Au* **2**, 1096-1104 (2022).
12. You, R. et al. NbO x /CeO_2 -rods catalysts for oxidative dehydrogenation of propane: Nb–CeO_2 interaction and reaction mechanism. *J. Catal.* **348**, 189-199 (2017).
13. Michorczyk, P., Kuśtrowski, P., Niebrzydowska, P. & Wach, A. Catalytic performance of sucrose-derived CMK-3 in oxidative dehydrogenation of propane to propene. *Appl. Catal., A: Gen.* **445-446**, 321-328 (2012).
14. Zhou, H. et al. Isolated boron in zeolite for oxidative dehydrogenation of propane. *Science* **372**, 76-80 (2021).
15. Ali, M. I., Sadatomi, M. & Kawaji, M. Adiabatic two-phase flow in narrow channels between two flat plates. *Can. J. Chem. Eng.* **71**, 657-666 (1993).
